# Investigation of Behavior and Plasma Levels of Corticosterone in Restrictive- and Ad Libitum-Fed Diet-Induced Obese Mice

**DOI:** 10.3390/nu14091746

**Published:** 2022-04-22

**Authors:** Martin Allweyer, Matthias Emde, Ina Bähr, Julia Spielmann, Philipp Bieramperl, Wiebke Naujoks, Heike Kielstein

**Affiliations:** Institute of Anatomy and Cell Biology, Faculty of Medicine, Martin Luther University Halle-Wittenberg, 06108 Halle, Germany; allweyer.martin@yahoo.de (M.A.); matthiasemde.1210@gmail.com (M.E.); ina.baehr@medizin.uni-leipzig.de (I.B.); julia.spielmann@uk-halle.de (J.S.); bieramperl.p@klinik-mallersdorf.de (P.B.); wiebke.naujoks@posteo.de (W.N.)

**Keywords:** obesity, behavior, mice, activity, anxiety, aggression

## Abstract

Diet-induced obesity (DIO) mice models are commonly used to investigate obesity-related health problems. Until now, only sparse data exist on the influence of DIO on behavior and stress hormones in mice. The present study investigates high-fat DIO with two different feeding regimes on behavioral parameters in mice. Various behavioral tests (open field, elevated plus maze, social interaction, hotplate) were performed with female BALB/c and male C57BL/6 mice after a feeding period of twelve weeks (restrictive vs. *ad libitum* and normal-fat diet vs. high-fat diet) to investigate levels of anxiety and aggression. BALB/c mice were DIO-resistant and therefore the prerequisite for the behavior analyses was not attained. C57BL/6 mice fed a high-fat diet had a significantly higher body weight and fat mass compared to C57BL/6 mice fed a control diet. Interestingly, the DIO C57BL/6 mice showed no changes in their aggression- or anxiety-related behavior but showed a significant change in the anxiety index. This was probably due to a lower activity level, as other ethological parameters did not show an altered anxiety-related behavior. In the *ad libitum*-fed DIO group, the highest corticosterone level was detected. Changes due to the feeding regime (restrictive vs. *ad libitum*) were not observed. These results provide a possible hint to a bias in the investigation of DIO-related health problems in laboratory animal experiments, which may be influenced by the lower activity level.

## 1. Introduction

Currently, ischemic heart diseases are globally the main cause of death [1]. Obesity is a severe risk factor for these and many more diseases. In 2016, worldwide, 39% of women and 39% of men aged > 18 years were overweight (body mass index (BMI) > 25 kg/m²) and 11% of men and 15% of women were obese (BMI > 30 kg/m²) [2]. Obesity is a major health problem and needs to be analyzed in detail. Due to the immense clinical relevance, many research projects, clinical trials, animal, and in vitro experiments are investigating different aspects concerning the health consequences of obesity. Different settings of obese mouse models exist. Genetic models display a molecular gene defect responsible for dysfunctional eating, energy metabolism, and/or autonomic activity. For example, the leptin pathway is altered in the *ob*/*ob* or *db*/*db* mouse or a GLUT4 (glucose transporter 4) overexpression is decisive for the highly elevated body weight in the GLUT4 transgenic mouse [3]. In rare cases, human obesity can be triggered by a monogenetic perturbation, too [4]. Nevertheless, in the vast majority of cases, obesity is a polygenetic and multifactorial cause health problem, including nutritional factors and reduced physical activity [5]. As a result, the diet-induced obesity (DIO) rodent model is commonly used to investigate obesity-related health problems [6].

Data published in recent years showed a large body of acquired knowledge in *ad libitum*-fed mice. However, less data about different patterns of feeding and the resulting changes in body weight, fat mass, metabolic parameters, or behavior can be found. Therefore, to improve the quality of DIO-based experiments, different feeding regimens and the resulting altered behavior of DIO mice have to be investigated [7,8]. Furthermore, it is necessary to evaluate the consequences of distinct feeding methods. An increasing number of scientific studies have examined the influence of fasting periods on processes of health and diseases [9,10].

Previous studies in obese mice showed an increased anxiety-like behavior and decreased sociability, as well as in their offspring [11,12]. In addition, recent studies have shown that obesity and stress are linked at the protein level, such as neuropeptide Y (NPY) or FK506-binding protein 51 (FKBP51). NPY stimulates proliferation and differentiation of new adipocytes and can increase the amount of abdominal fat, resulting in a metabolic syndrome-like condition. Furthermore, it could be shown that the hypothalamic FKBP51 expression is responsive to diet and stress, and a higher basal expression of this protein was related to an increased body weight gain [13,14]. Nevertheless, less data exist objectifying an obesity-induced alteration of stress levels in DIO mice and the relationship to an altered behavior.

In humans, epidemiological data observing the interaction between stress in different variations, adiposity, adiposity-based chronic diseases, and the reactivity of the hypothalamic-pituitary-adrenal axis, exist [15,16,17,18]. Corticosterone is one mediator of stress and can be used to objectify the stress level [19,20]. It is essential to characterize DIO mice models concerning their general and specific behavioral patterns of anxiety and aggressiveness in order to adapt the researcher’s handling and the experimental environment on the altered behavior. This is necessary to reduce the stress level of the mice and therefore to avoid an influence on the experimental procedures. For example, experiments examining the relationship between obesity and psychiatric illness may require a special stress reduction protocol to be established if obese mice are more vulnerable to stress. Furthermore, it can help to enhance our understanding of further obesity-driven disorders.

The present paper investigates the effects of DIO caused by a high-fat diet with different feeding methods on behavioral patterns and corticosterone plasma levels in two different mouse strains, female Balb/c and male C57BL/6 mice. We hypothesized that DIO mice exhibit higher levels of anxiety and aggression, and that this behavior will be more pronounced in *ad libitum*- than in restrictive-fed mice.

## 2. Materials and Methods

### 2.1. Animals

Six-week-old female BALB/c mice (*n* = 28) and male C57BL/6 mice (*n* = 36) from the Charles River Laboratories (Sulzfeld, Germany) were used. All animals were housed in single plastic-based cages equipped with standard bedding, a small red house, and a piece of paper to build their own nest. The strains were separated in their own pathogen-free climate chamber under controlled conditions at 23 ± 2 °C and 55% ± 5% relative humidity, and a sound-proof locker under a dark/light cycle from 12 to 12 h with lights on at 07:00 a.m. Prior to the start of the experiment, mice were acclimatized and handled daily according to a protocol for a period of one week. During the feeding period, all mice were handled weekly. All research and animal care procedures have been approved by the local Animal Care Committee (“Landesverwaltungsamt Halle”, reference number 42502-2-1341 MLU).

### 2.2. Diets and Feeding Regimens

Each of the two different mice strains was randomized to a normal-fat diet (NFD), or to induce obesity, in a high-fat diet (HFD) group. For the BALB/c strain, this resulted in an equal division of 14 mice per group. The C57BL/6 mice were divided into 14 mice in the control group and 22 mice in the DIO group, as we anticipated having possible non-responders in this strain in the DIO group [21]. In the next step, these four groups were equally separated into an *ad libitum*- and a restrictive-fed group. At the beginning of the dark phase, the restrictive-fed animals received 90% of the consumed food from the corresponding *ad libitum* group from the day before. Diets were received from Research Diets, Inc. (New Brunswick, NJ 08901 USA). The control groups were fed the D12450Ji diet (10% kcal fat content) and the DIO groups were fed the D12492 high-fat diet (60% kcal fat content). In order to prevent deficiencies in dietary components and energy, the necessary daily intake was determined using the manufacturer’s dietary composition information and the daily dietary intake. Water was available *ad libitum* throughout the whole experiment for all participating groups.

**Figure 1:**
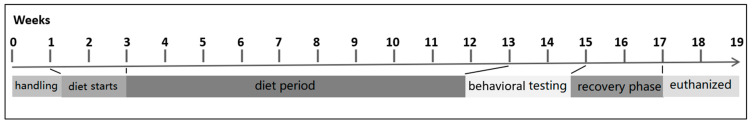
Timeline.

Food intake was weighed daily at the same time and the body weight was documented on the same day each week. The cages were changed once a week while weighing the animals in order to cause as little stress as possible. Seventeen weeks after the start of the diet phase and two weeks after the completion of the behavioral tests, mice were euthanized. The timeline is illustrated in Figure 1.

### 2.3. Behavioral Tests

Behavioral tests started twelve weeks after the initiation of the diet. One test per day was performed and each strain was examined on different days, except for the hotplate test, where both strains were tested in parallel. All tests were started one hour after the start of the dark cycle. With the exception of the hotplate test, all tests were recorded with a camera (VIDO B/W CCD Camera Au-CB602, VIDO AT, Vienna, Austria) and analyzed with the Viewer III software (Biobserve GmbH, Bonn, Germany). The program determines the head, body, and tail of the mouse. The time in a certain area is counted as soon as the center of the body is in the respective area. Considering the different coat color of both strains, the arenas were colored using a white adhesive tape for the C57BL/6 mice and a black one for the BALB/c mice, receiving a marked contrast of the mice in reference to the background. A red electric lighting was used while performing the experiments since mice react less sensitively to long-wavelength red light [22]. All experiments were performed by the same two researchers blind to the receiving diet and feeding regime.

#### 2.3.1. Open Field (OF)

The OF test was used to detect anxiety and agitation [23]. The device used consisted of a quadratic wooden board of 65 × 65 cm and 30 cm wooden walls. At the beginning of the experiment, one mouse was placed between two corners with direction of view towards the center of the arena. The test was recorded for five minutes. The following parameters were measured: center time, corner time, track length, wall distance, rearing, pile of feces, and the center distance/total distance ratio as an anxiety index.

#### 2.3.2. Elevated Plus Maze (EPM)

The EPM was applied to detect anxiety [24,25]. The device consists of a 51.3 cm-high wooden maze. The dimensions of the open arms were 30 cm of length to 5 cm of width, and a small 0.5 cm-high edge. The dimensions of the closed arms were 30 cm of length to 5 cm of width, and a 15 cm-high wall. At the beginning, the mouse was placed in the middle field facing an open arm, and the middle field was defined as a separate area. The test was recorded for five minutes. The first analyses for all groups started with the following parameters: number of total entries, number of closed-arm entries, percent of open-arm entries (vs. total number of entries), and percent of open-arm time (vs. total tested time). To enhance the level of validity, a second analysis was performed, including the following ethological parameters: rearing, percent of protected head dip, amount of total head dip, and number of piles of feces. Protected head dip is a risk assessment behavior. The mouse leans from the center or closed-arm area forward to the center or open-arm area. It can be used to detect an anxiogenic effect even in the absence of changes in conventional open-arm measurement [24,25,26].

#### 2.3.3. Social Interaction (SI)

The SI test can be used to detect anxiety, aggressiveness, and nosiness. The test was performed in the same arena as the OF test. For each run, two mice of the same experimental group were randomly paired. The mice were placed in two opposite corners of the field. The experiment was recorded for ten minutes. We focused on aggressive-like behavior and measured numbers of bites and fighting.

#### 2.3.4. Hotplate (HP)

The HP test measures the nociceptive reflexes on peripheral, medullar, and brain levels. All levels of these must be intact to notice pain. In the setting of the present study, the mouse was placed in the middle of the plate (Hotplate 602001; TSE Systems), heated at a temperature of 53 ± 0.5 °C. Time to elicit jumping or paw licking was measured. The cut-off time was 30 s to prevent tissue damage [27]. We included this test as a marker for Polyneuropathy. It was hypothesized that Polyneuropathy could be a possible confounder if a mouse developed some kind of dizziness due to an afferent disorder.

### 2.4. Measurement of Plasma Corticosterone Concentration

Two weeks after the completion of the behavioral tests and seventeen weeks after the beginning of the diet, mice were euthanized by puncture of the right heart ventricle under an isoflurane anesthesia to collect blood samples. To analyze the plasma corticosterone concentrations, the DirektX corticosterone chemiluminescence immunoassay kit from Arbor Assays (Ann Arbor, MI, USA, Catalog Number K014-C1) was used by following the manufacturer’s instructions.

### 2.5. Statistics

Data are expressed as means ± standard error of the mean (SEM) and were analyzed by SPSS (IBM, Armonk, NY, USA), using two-way ANOVA with the main factors, “diet” and “feeding regime”, and with the post hoc Tukey’s multiple comparison test. Since the feeding regimes did not induce any altered behavior, we combined the DIO and control groups to facilitate the graphic representation (only in the OF and EPM). These groups were analyzed by a *t*-test. Differences were considered significant if *p* < 0.05.

## 3. Results

One mouse in the restrictive control group of the BALB/c mice died during the feeding period—the reason was unknown.

### 3.1. Body Weight

**Figure 2:**
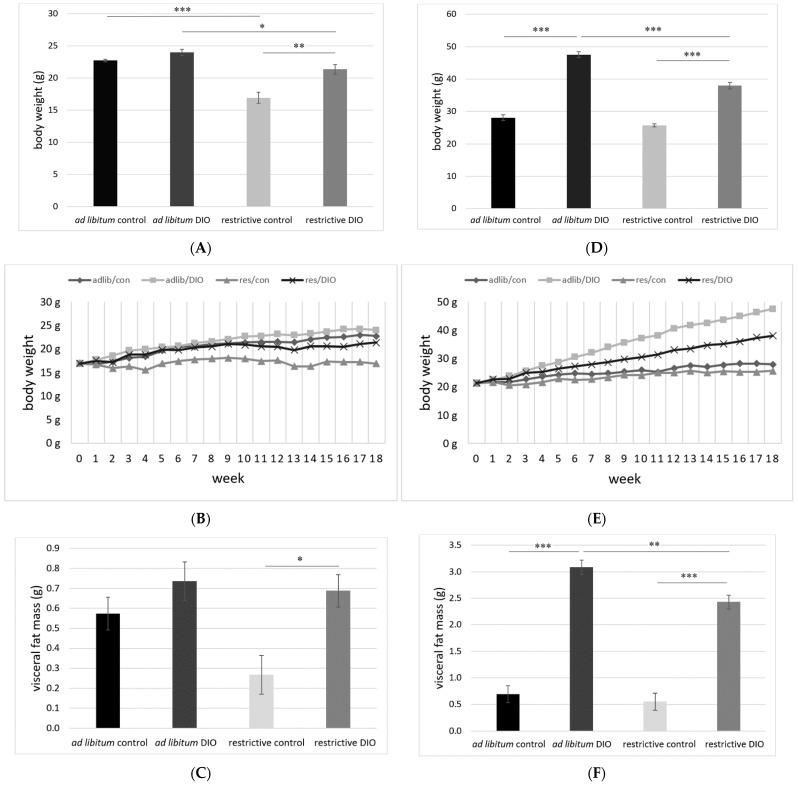
(**A**) Final body weights in BALB/c mice. (**B**) Body weight gain in BALB/c mice. (**C**) Visceral fat mass in BALB/c mice. (**D**) Final body weights in C57BL/6 mice. (**E**) Body weight gain in C57BL/6 mice. (**F**) Visceral fat mass in C57BL/6 mice. g = gram, adlib = *ad libitum*, res = restrictive, con = normal weight DIO = diet-induced obesity. Means ± standard error of mean (SEM), * *p* ≤ 0.05, ** *p* ≤ 0.01, *** *p* ≤ 0.001.

Figure 2A shows the final body weights of BALB/c mice. The restrictive control group had the lowest body weight and differed significantly from all other groups. The final body weight of the restrictive DIO group also differed significantly compared to the *ad libitum* DIO. The normal body weight of 12-week-old BALB/c mice is 23 g (range 21–25.3 g), with a weight gain similar to the weight gain in our mice [28,29]. With the exception of the restrictive control group, all other BALB/c mice reached a normal body weight (Figure 2A). With a mean body weight of 17 g (Figure 2B) and a visceral fat mass of 0.27 g (Figure 2C), the restrictive control group was underweight.

Figure 2D shows the final body weight of C57BL/6 mice. A significant difference between both *ad libitum*, both restrictive, and both DIO groups could be detected, but not between the control groups. The DIO groups markedly increased their body weight, whereas the control groups showed a normal body weight. Figure 2E demonstrates a continuous body weight gain of both DIO C57BL/6 groups, whereas the control groups did not markedly increase their body weight over the feeding period. As a marker for a successful diet-induced obesity, both DIO groups had a significantly higher absolute visceral fat mass (*ad libitum* 3.1 g; restrictive 2.4 g) compared to the control groups (*ad libitum* 0.7 g; restrictive 0.6 g) (Figure 2F).

The influence of the diet as well as the feeding regimen (restrictive vs. *ad libitum*) was more pronounced in C57BL/6 male compared to BALB/c female mice.

The prerequisite for the behavioral testing was diet-induced obesity to analyze changes of aggression and anxiety-like behavior due to this factor. In addition, underweight mice show changes in their behavioral patterns, e.g., activity [30,31,32]. Due to these facts, we could not include the BALB/c mice in the behavioral analyses, and therefore the behavioral data shown in the present study originate from the C57BL/6 mice.

### 3.2. Open Field (OF)

**Figure 3:**
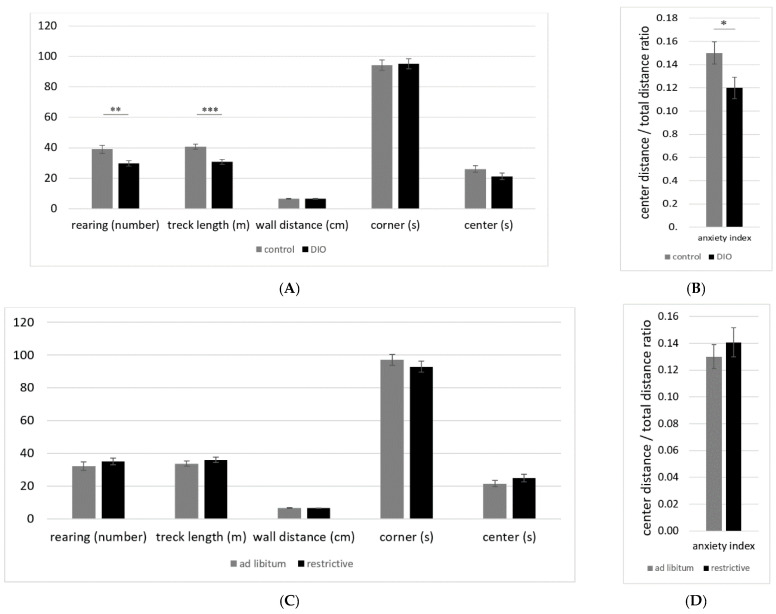
(**A**) Open-field test with C57BL/6 mice, control = normal weight, DIO = diet-induced obesity, means ± standard error of mean (SEM), ** *p* ≤ 0.01, *** *p* ≤ 0.001. (**B**) Anxiety index (center distance/total distance ratio) of C57BL/6 mice, control = normal weight, DIO = diet-induced obesity, means ± standard error of mean (SEM), * = *p* ≤ 0.05. (**C**) Open-field test with C57BL/6 mice. (**D**) Anxiety index (center distance/total distance ratio) of C57BL/6 mice.

In the OF test, the DIO C57BL/6 mice showed a significant decrease in incidents of rearing, a significantly shorter track length (Figure 3A), and additionally a significantly lower anxiety index (center distance/total distance ratio; Figure 3B). No differences could be observed concerning wall distance, center, and corner time. The difference in the anxiety index is most likely a result of the lower track length and inactivity of the DIO groups, since wall distance, center, and corner time were not significantly altered compared to the normal-weight control animals. No significant differences or trends suggesting an alteration of the anxiety-related behavior due to the feeding regime could be observed (Figure 3C,D).

### 3.3. Elevated Plus Maze (EPM)

**Figure 4:**
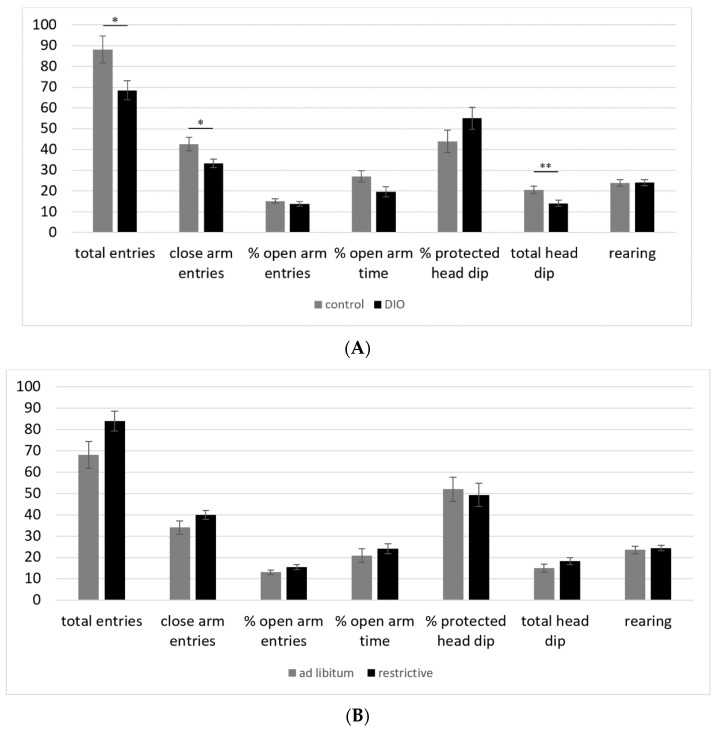
(**A**) Elevated plus maze test with C57BL/6 mice, control = normal weight, DIO = diet-induced obesity, means ± standard error of mean (SEM), * *p* ≤ 0.05, ** *p* ≤ 0.01. (**B**) Elevated plus maze test with C57BL/6 mice, means ± standard error of mean (SEM).

DIO C57BL/6 mice showed a significantly lower number of total and closed arm entries (Figure 4A). Additionally, only a significantly lower number of total head dips was observed. We noticed that the control group moved frequently between the central zone and the closed arm without entering the open arm. It is therefore highly probable that these results represent a lower level of activity in DIO compared to normal-weight control mice and do not show a different anxiety level. In addition, the pile of feces also shows a significant difference between the DIO group, with a mean value of 0.14, and the control group, with a mean value of 0 pile of feces (*p* = 0.01), and this minimal difference could also be a result of the amount of feeding. Differences due to the feeding regime could not be observed (Figure 4B).

### 3.4. Social Interaction (SI)

No fighting events could be observed in the SI test. Bites were observed 0.57 times per mouse in the C57BL/6 restrictive and *ad libitum* control groups. In the DIO groups, bites could be observed in the restrictive group 0.36 times per mouse and in the *ad libitum* group, 0.2 times per mouse. However, neither the diet nor the feeding regimen had a significant influence on the biting events.

### 3.5. Hotplate (HP)

**Figure 5:**
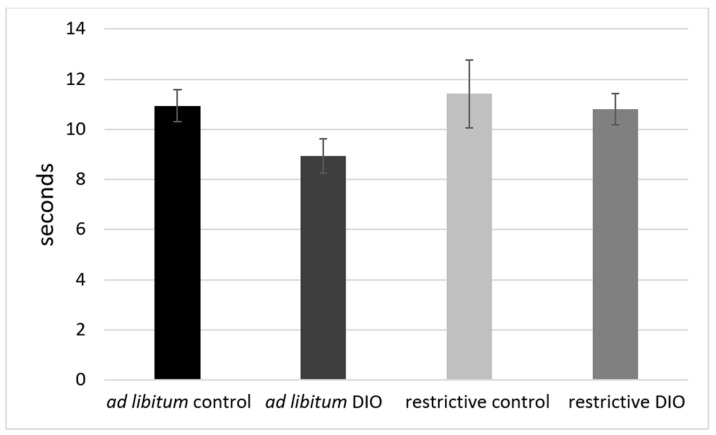
Hotplate test with C57BL/6 mice. DIO = diet-induced obesity, means ± standard error of mean (SEM).

In terms of the nociceptive reflexes, no effect of the diet or the feeding regimen could be observed. The *ad libitum*-fed DIO group showed the earliest reaction after 9 s, possibly due to the increased body weight and the resulting increased pressure on the hotplate (Figure 5).

### 3.6. Plasma Corticosterone Concentration

**Figure 6:**
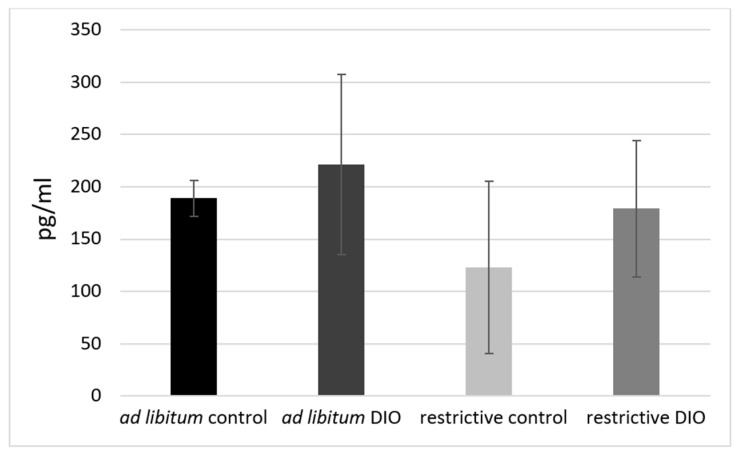
Plasma corticosterone levels in C57BL/6 mice. DIO = diet-induced obesity, means ± standard error of mean (SEM).

The hormone values of all groups were relatively low as compared to other studies [33,34]. Although the *ad libitum*-fed DIO group showed the highest corticosterone level and the restrictive control group showed the lowest corticosterone level, these results were not significant (Figure 6).

## 4. Discussion

To investigate the pathophysiology of obesity, different DIO mouse models are widely used. However, only very little information exists concerning possible behavioral changes (e.g., altered stress levels, anxiety, or aggression) due to the dietary intervention. Previous studies demonstrated that DIO F344 rats develop a significantly higher level of anxiety- and aggression-related behavioral parameters compared to their normal-weight littermates [35].

In the present study, two mice strains (female BALB/c and male C57BL/6) were examined. Furthermore, the use of two different feeding regimens (restrictive and *ad libitum*) was implemented. Comparable to other studies, the body weight gain in HFD-fed BALB/c mice compared to their corresponding NFD groups was negligible, compared to the significantly higher body weights in both DIO C57BL/6 mice groups compared to their corresponding control groups [36]. A dietary response rate in BALB/c mice of about 66% and an interpretation as an obese-resistant mouse strain has already been described [37,38]. It could be shown that one possible mechanism depends on the Nod2 protein, which is a pattern-recognition receptor in the primary immune system with influence on the microbiome [39]. Although there are tendencies for an increased visceral fat mass between the *ad libitum* control group and the *ad libitum* DIO group in BALB/c mice, no significant difference could be detected (Figure 2C). Future studies should examine the total fat mass to determine possible significant differences at similar body weights, as demonstrated for female C57BL/6 mice [40].

In order to detect anxiety in various specifications, different behavioral tests were performed and ethological parameters were investigated, especially in the open-field and in the elevated plus maze tests [24,25,41]. Results of the present study demonstrate that the differences between normal-weight control and DIO C57BL/6 animals were not solely caused by an altered anxiety level, but predominantly by a significantly lower level of activity—most likely as a consequence of the higher body weight and associated physical restrictions. There are several possible ways how the body weight could influence the level of activity. For example, it can affect the cardiovascular system and thereby increase blood pressure through the renin-angiotensin system, which could affect endurance [42]. In addition, obesity is an independent and modifiable risk factor for osteoarthritis. It influences the pathophysiology of biomechanical and inflammatory factors [43]. Furthermore, specific experimental setups can be demanding for obese mice, such as the open arms of the elevated plus maze. It can be a great challenge for these mice to turn around and keep their balance. In addition, it is well-known that leptin levels are increased in obese mice and humans [44], resulting in reduced concentration of the hypothalamic neuropeptide Y (NPY) [45]. NPY-deficient mice show a lower level of activity [46]. Thus, this may be a possible mechanism as to how obesity can influence the locomotor activity, both in mice and in humans. However, studies also exist demonstrating unaffected activity parameters in obese rodents. Brownlow et al. could not find altered activity levels between high-fat/high-sucrose-, high-fat/low-sucrose-, low-fat/high-sucrose-, or low-fat/low-sucrose-fed C57BL/6 mice (mean body weight differed from 24 to 40 g) [47]. Nevertheless, a review from 2017 showed that DIO mice and rats show an overall decrease in the amount of activity during the night and a circadian disruption of feeding behavior [48]. The body weight of the C57BL/6 mice in the present study was strongly increased, which may explain the significantly altered activity level. These differences were probably unaffected by restrictive feeding. Restrictive feeding is used as a model for activity-based anorexia, as some normal-weight mouse strains show increased activity levels under restrictive feeding, but not C57BL/6 mice [31,32]. We also found no effect on the activity level due to the feeding regime.

This knowledge is important for activity-dependent studies with mice, e.g., the water maze. Obese mice may need more time to reach the platform, not because of a lower sense of direction, but because of their increased weight. Furthermore, this result of the present study is also of importance for studies investigating the relationship between bone architecture and obesity [49], since both diet and activity affect the bone properties.

So far, no study exists investigating aggression in DIO mouse models. However, previous studies showed a significantly higher level of aggression in DIO F344 rats compared to their normal-weight littermates [35]. In contrast, the present study could not detect any differences concerning aggressive-like behavioral parameters (e.g., fighting or biting) in the SI test. Although the SI test is an appropriate tool to measure aggression-like behavior, future studies should investigate potential aggressive behavior with other experimental settings, e.g., in neutral vs. home cage areas, including different housing conditions prior to testing [35,50]. Individual housing of mice promotes aggression in the social interaction test, and thus the housing conditions were suitable for testing aggression behavior [51,52]. Differences in activity levels between individually and group housed mice are known [53]. However, since the conditions were the same for all groups, a significant influence of this factor on the reported differences can be neglected. Nevertheless, it should be investigated whether housing conditions have different effects on normal-weight and DIO mice. It should be considered that group-housed mice can develop competitive behavior, and therefore the food intake of the individual mice can be different compared to a littermate of the same experimental group.

DIO can induce type II diabetes in C57BL/6 mice and thus be causative for the development of diabetic Polyneuropathy [54]. In the present study, none of the experimental animals showed clinical signs of diabetes nor ataxia as an indirect sign for a diabetic Polyneuropathy. Comparable with other results, our study failed to show any change of the acute thermal pain perception in DIO mice in the HP test [55,56]. Thus, Polyneuropathy as a disruptive factor for activity is very unlikely.

The highest corticosterone level was detected in the *ad libitum* DIO group with the highest body weight, without any significant difference between the investigated groups. Other studies showed significantly higher corticosterone levels in obese mice [34,57]. However, these studies have measured higher corticosterone levels in a ng/mL range. A measurement error was largely ruled out after multiple controls of the data. Based on these data, there is no indication of a generally increased stress level in the DIO mice. It has to be taken into consideration that it is challenging to compare corticosterone levels between different studies because of various factors possibly influencing the plasma levels (daytime, housing condition, temperature, handling before euthanization, etc.) [58,59]. Nevertheless, it is important to investigate corticosterone in DIO mice models since it is well-known that corticosterone influences the fat distribution, the development of obesity, the metabolic syndrome, cardiovascular diseases, and probably also psychiatric disorders in humans [15,60,61].

In the present study, the moderate feeding restriction of 90% in C57BL/6 mice had no influence on the behavioral pattern itself. Nevertheless, it influenced the body weight in the high-fat groups compared to the normal-weight groups. It must be assumed that the changes in the activity level were most probably caused by the higher body weight and not by the way the mice were fed (*ad libitum* vs. restrictive).

A limitation of this study is the use of female BALB/c and male C57BL/6 mice, making comparisons difficult due to the two different perspectives: strain and sex. Therefore, the results presented here can solely be assigned to male C57BL/6 or female BALB/c mice. Further studies should attempt to implement both sexes. Furthermore, in future behavioral studies with DIO mice, leptin and NPY should be determined to better understand the physiology of altered activity levels. An increased leptin level could already be detected in DIO mice, but without a direct connection to a changed behavior [62].

## 5. Conclusions

The present study showed a significantly lower activity level, a decreased anxiety index, and unchanged aggressive-like behavior in male DIO C57BL/6 mice. Behavioral alterations in obese experimental animals should be kept in mind when analyzing results of immunological, metabolic, endocrinological, or psychiatric experiments.

## Data Availability

Not applicable.
